# Whole-Genome Methylation Analysis of Phenotype Discordant Monozygotic Twins Reveals Novel Epigenetic Perturbation Contributing to the Pathogenesis of Adolescent Idiopathic Scoliosis

**DOI:** 10.3389/fbioe.2019.00364

**Published:** 2019-12-10

**Authors:** Gang Liu, Lianlei Wang, Xinyu Wang, Zihui Yan, Xinzhuang Yang, Mao Lin, Sen Liu, Yuzhi Zuo, Yuchen Niu, Sen Zhao, Yanxue Zhao, Jianguo Zhang, Jianxiong Shen, Yipeng Wang, Guixing Qiu, Zhihong Wu, Nan Wu

**Affiliations:** ^1^Department of Orthopedic Surgery, Peking Union Medical College Hospital, Peking Union Medical College and Chinese Academy of Medical Sciences, Beijing, China; ^2^Beijing Key Laboratory for Genetic Research of Skeletal Deformity, Beijing, China; ^3^Institute of Biomedical Big Data, Wenzhou Medical University, Wenzhou, China; ^4^Department of Central Laboratory, Peking Union Medical College Hospital, Peking Union Medical College and Chinese Academy of Medical Sciences, Beijing, China; ^5^Medical Research Center of Orthopedics, Chinese Academy of Medical Sciences, Beijing, China

**Keywords:** adolescent idiopathic scoliosis (AIS), monozygotic twins, whole exome sequencing (WES), DNA methylation, whole genome bisulfite sequencing (WGBS)

## Abstract

**Background:** Adolescent idiopathic scoliosis (AIS) is a complex disease affecting a large number of teenagers, especially in female. This study reveals novel epigenetic perturbation to the pathogenesis of AIS.

**Methods:** A female monozygotic (MZ) twin pair discordant for AIS were examined for whole-exome sequencing and epigenome difference. Sets of differentially methylated regions (DMRs) were validated using MethylTarget™ method in 20 AIS female patients and 20 healthy female controls.

**Results:** Few exome difference but several potential DMRs were found between the MZ twins. We identified 313 hypermethylated DMRs and 397 hypomethylated DMRs, respectively. Most of them were enriched in the MAPK and PI3K-Akt signaling pathway, which may contribute to the discordance of AIS. Several DMRs related to scoliosis genes were tested, and the *NDN*: TSS-DMR (chr15:23932133-23932304, hg19) was confirmed in additional samples. The methylation level of this DMR was significantly higher in the AIS group than in the control group (*p* = 0.04).

**Conclusions:** We described the epigenome difference in an AIS female discordant MZ twin pair using Whole Genome Bisulfite Sequencing (WGBS). The *NDN*: TSS-DMR had higher methylation level in female AIS, which can help elucidate the potential etiology of AIS.

## Introduction

Adolescent idiopathic scoliosis (AIS) is a 3-dimensional spinal deformity affecting 1–3% of children in the world (Yawn et al., 1999). Although studies to discover the underlying mechanisms of AIS had been conducted for many years, the etiology is still unclear. Previous studies showed that genetic factors from single nucleotide variant (SNV) to copy number variants (CNV), played a pivotal role in the development of AIS (Kesling and Reinker, 1997; Li et al., 2016; Gao et al., 2017). Based on genome-wide association study (GWAS) and other similar association studies, single nucleotide polymorphisms (SNPs) of several genes such as *GPR126* (Kou et al., 2013; Liu et al., 2018), *PAX1* (Sharma et al., 2015; Liu et al., 2019), *LBX1* (Takahashi et al., 2011; Liu et al., 2017), and *BNC2* (Ogura et al., 2015), were known to increase the risk of AIS. There was also evidence that rare mutations of some genes may be responsible for AIS, such as *FBN1* (Buchan et al., 2014a), *AKAP2* (Li et al., 2016) and *MAPK7* (Gao et al., 2017). Other studies illustrated the association between CNV and AIS (Buchan et al., 2014b). All of those genetic factors can only explain the etiology of 2–7.6% of AIS (Buchan et al., 2014a; Ikegawa, 2016).

The abnormal, or aberrant DNA methylation pattern is universally recognized as an important factor in diseases, especially complex diseases. Recent studies indicated that differential methylation of key genes or CpG site were related to AIS, such as site cg01374129 (Meng et al., 2018), *COMP* (Mao et al., 2018), and *PITX1* (Shi et al., 2018). But there were few types of research focusing on the methylation status of AIS on the whole-genome scale and no research of monozygotic twins discordant for AIS.

Monozygotic (MZ) twins are outstanding subjects to analyze epigenetic mechanisms. Theoretically, they share almost the same genotype, but the epigenome could be different, especially for discordant MZ twins who display phenotypically discordant on disease traits. Therefore discordant MZ twins are analyzed for deciphering complex diseases, such as congenital heart disease (Lyu et al., 2018), type 1 diabetes (Elboudwarej et al., 2016), and congenital renal agenesis (Jin et al., 2014).

In this study, we enrolled a MZ female twin pair discordant for AIS from Chinese Han population. In this twin pair, one was diagnosed with AIS, but her twin sister was healthy and had no spinal deformity. Their parents had no abnormality of the skeletal system or related family history. We compared their exome variants and genome methylation difference aiming to find the contributions to the pathogenesis of AIS.

## Materials and Methods

### Patients and Materials

Monozygotic twin discordant for AIS was recruited from Peking Union Medical College Hospital (PUMCH). There was no consanguinity between the parents. The parents and monozygotic twin of the patient were healthy without abnormalities of the skeletal system. Twenty sporadic AIS female patients and 20 healthy female controls were enrolled as a replication cohort. Genomic DNA was extracted from their peripheral blood using DNeasy Blood & Tissue Kits (QIAGEN, Eastwin Scientific, Inc., Beijing, China) according to the manufacturer's instructions. This study was approved by the Ethical Review Board of Peking Union Medical College Hospital. Written informed consent was obtained from all the participants or their parents.

### Whole Exome Sequencing (WES) and Analysis

WES was performed on DNA extracted from the peripheral blood. In brief, libraries were prepared from DNA samples and subjected to whole-exome capture using the VCRome SeqCap EZ Chice HGSC 96 Reactions capture reagent (Roche), followed by sequencing on an Illumina HiSeq 4000 platform with 150-bp pair-end reads mode. Whole-exome sequencing for all individuals in this family was performed and each generated fully reads with a mean depth of 107 (Table S1).

The variant-calling and annotation were performed by the in-house developed Peking Union Medical college hospital Pipeline (PUMP), same as in our previous studies (Wang et al., 2018a,b). In brief, single-nucleotide variants and internal duplications and/or deletions (aka indels) were called using the HaplotypeCaller of the Genome Analysis Toolkit (v3.4.0). Annotation of the *de novo*, compound heterozygotes, and recessively inherited variants was calculated with Gemini (v0.19.1) for *in silico* subtraction of parental variants from the proband's variants, with accounting for read number information extracted from BAM files. Computational prediction tools Gerp++ (Davydov et al., 2010), CADD (Kircher et al., 2014), SIFT (Vaser et al., 2016), Polyphen-2 (Adzhubei et al., 2010), and MutationTaster (Schwarz et al., 2014) were used to predict the conservation and pathogenicity of candidate variants. All variants were compared against Deciphering Disorders Involving Scoliosis & Comorbidities (DISCO) study in-house database and publicly available databases including the 1000 Genomes Project (http://www.internationalgenome.org/), the Exome variant server, NHLBI GO Exome Sequencing Project (ESP) (http://evs.gs.washington.edu/EVS/), and the Exome Aggregation Consortium (ExAC) (http://exac.broadinstitute.org/).

After data annotation, we first identified *de novo* mutations in the AIS patient taking her parents as a reference and then comparison was made between the MZ twins. For the mutations between the MZ twins including SNVs and short insertions/deletions (InDels), we classified them into four types (Figure S1). In the Equal type, both of the twins had the same mutations comparing to the Reference Genome. In the different type1, only the case had the mutation. In the different type2, the mutations only presented in the Control. In the different type3, both of the twins had different mutation compared to the Reference Genome. We hypothesized that the different type1 and type3 were potentially pathogenic.

### Whole Genome Bisulfite Sequencing (WGBS)

#### Library Preparation and Quantification

A total amount of 5.2 micrograms genomic DNA spiked with 26 ng lambda DNA were fragmented by sonication to 200–300 bp with Covaris S220, followed by end repair and adenylation. Cytosine-methylated barcodes were ligated to sonicated DNA as per manufacturer's instructions. Then these DNA fragments were treated twice with bisulfite using EZ DNA Methylation-GoldTM Kit (Zymo Research). And the resulting single-strand DNA fragments were PCR amplificated using KAPA HiFi HotStart Uracil + ReadyMix (2X).

Library concentration was quantified by Qubit^®^ 2.0 Fluorometer (Life Technologies, CA, USA) and quantitative PCR, and the insert size was checked on Agilent Bioanalyzer 2100 system.

#### Clustering and Sequencing

The clustering of the index-coded samples was performed on a cBot Cluster Generation System using TruSeq PE Cluster Kit v3-cBot-HS (Illumia) according to the manufacturer's instructions. After cluster generation, the library preparations were sequenced on Illumina Hiseq 2500 platform and 100 bp single-end reads were generated. Image analysis and base calling were performed with the standard Illumina pipeline, and finally, 100 bp paired-end reads were generated.

### WGBS Data Analysis

Raw WGBS reads were mapped to the human reference genome (hg19, NCBI build 37.1) using BSMAP v2.90 (default parameters). Adaptor, low-quality and duplicated reads were automatically trimmed by BSMAP v2.90 with default thresholds. Single base methylation ratio was measured as the proportion of methylated Cs in all mapped reads from both strands. Only CpG sites with covered reads ≥4 reads were considered for further analysis.

Differentially methylated regions (DMRs) between the twin pair were identified using MethPipe 3.4.3 with significant differentially CpGs ≥ 5 and a minimal number of 10 CpGs that the DMR spans. DMRs were classified to “hypermethylation group” and “hypomethylation group” based on methylation level difference and these regions were ranked by absolute methylation difference between scoliosis and normal sample in the MZ twin (Figure S2). Microarray probes were designed for DMRs from the twin pair based on physical location, the methylation state of each interval was quantified by the mean of methylation beta values. Pearson correlation score was used to estimate the reliability of WGBS data.

### Annotation of DMRs Associated Genes

Genomic position distribution of DMRs was performed using ChIPseeker (v1.14.0) Bioconductor package. Every DMR starting from 3 kb downstream to 3 kb upstream of the TSS was assigned to the corresponding RefSeq gene. Functional enrichment analysis of DMRs associated genes at KEGG pathways was performed using clusterProfiler (v3.6.0) Bioconductor package.

### Enrichment With Scoliosis Related Genes in DMRs

We searched public database including HPO, OMIM, Clinvar, and Pubmed using scoliosis as key term, 940 associated genes were collected as a scoliosis related gene set (Table S2). For these genes, an enrichment *P*-value was calculated based on hypergeometric testing:F

$$\begin{array}{l} P=\frac{C_{M}^{x}C_{N-M}^{k-x}}{C_{N}^{k}},\;k\in \left\{0,\;1,\;2,\ldots ,m\right\} \end{array}$$

*N* represents the total gene counts of RefSeq database, *M* means the total number of scoliosis-related genes, *k* is the number of genes that have mutations identified from WES data, and *x* is the number of intersected genes between these two gene sets.

For the 940 scoliosis related genes, an enrichment *P*-value was also calculated based on hypergeometric testing above. Here *N* is total RefSeq genes (27967), *M* is the number of scoliosis-related genes, *k* is the number of DMRs associated genes, and *x* is the number of intersected genes between the two gene sets.

### Validation in the Replication Cohort

#### Bisulfite Conversion and Multiplex Amplification

DNA methylation level was analysis by MethylTarget^TM^ (Genesky Biotechnologies Inc., Shanghai, China), an NGS-based multiple Targeted CpG methylation analysis method. Specifically, the genomic regions of interest were analyzed and transformed into bisulfite-converted sequences by geneCpG software. PCR primer sets were designed with the Methylation Primer software from bisulfate converted DNA.

Genomic DNA (400 ng) was subjected to sodium bisulfite treatment using EZ DNA Methylation™-GOLD Kit (Zymo Research) according to the manufacturer's protocols. Multiplex PCR was performed with optimized primer sets combination. A 20 μl PCR reaction mixture was prepared for each reaction and included 1x reaction buffer (Takara), 3 mM Mg^2+^, 0.2 mM dNTP, 0.1 μM of each primer, 1U HotStarTaq polymerase (Takara) and 2 μl template DNA. The cycling program was 95°C for 2 min; 11 cycles of 94°C for 20 s, 63°C for 40s with a decreasing temperature step of 0.5°C per cycle, 72°C for 1 min; then followed by 24 cycles of 94°C for 20 s, 65°C for 30 s, 72°C for 1 min; 72°C for 2 min.

#### Index PCR

PCR amplicons were diluted and amplified using indexed primers. Specifically, a 20 μl mixture was prepared for each reaction and included 1x reaction buffer (NEB Q5^TM^), 0.3 mM dNTP, 0.3 μM of F primer, 0.3 μM of index primer, 1 U Q5^TM^ DNA polymerase (NEB) and 1 μL diluted template. The cycling program was 98°C for 30 s; 11 cycles of 98°C for 10 s, 65°C for 30 s, 72°C for 30 s; 72°C for 5 min. PCR amplicons (170bp-270bp) were separated by agarose electrophoresis and purified using QIAquick Gel Extraction kit (QIAGEN).

#### Sequencing

Libraries from different samples were quantified and pooled together, followed by sequencing on the Illumina MiSeq platform according to manufacturer's protocols. Sequencing was performed with a 2 × 150 bp paired-end mode.

#### Data Analysis

Quality control of sequencing reads was performed by FastQC. Filtered reads were mapped to genome by Blast. After reads recalibration with USEARCH, methylation and haplotype were analyzed using Perl script. Statistics were performed by *U*-test and ANOVA.

## Results

### Clinical Information

**Figure 1:**
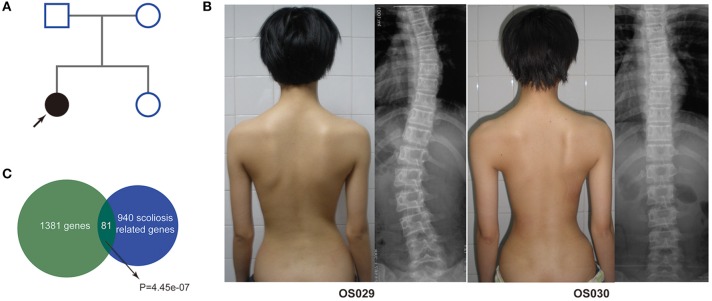
Clinical and WES findings of the AIS discordant monozygotic twin. **(A)** Pedigree of the patient. **(B)** Clinical characteristics and imaging of the proband and her healthy twin sister*. **(C)** Enrichment of WES mutation genes with scoliosis related genes. *Written informed consent was obtained from their parents for the publication of this image.

The monozygotic twins were 15-year-old girls, one of them (OS029) was diagnosed with AIS at 12. The patient had two apexes of scoliosis, T3-T11 with Cobb angle of about 27 degrees and T12-L3 with Cobb angle of about 26 degrees. The spine images of the healthy sister (OS030) and parents were normal (Figures 1A,B). In the replication cohort, the mean age of the AIS patients was 15 years old, and the mean age of the controls was 28 years old. All of the participants were Chinese females.

### Whole-Exome Sequence Results

After strict filtering and genomic annotation, we failed to identify pathogenic variants responsible for the discordant phenotype in the MZ twin. Finally, only three *de novo* variants were identified, one missense SNV in the exon of *PASD1* (c.425C>T), one missense SNV in the exon of *SLC44A4* (c.1281G>C) and one synonymous SNV in the exon of *AGTR2* (c.1080C>A), but all the *de novo* exome variants occur in both MZ twins (Table S3). Then we compared all the exome variants between the MZ twins. There were 30,700 same mutations, 32 Type 1 mutations and 1,830 Type 3 mutations. There were no Type 2 mutations (Table 1). Altogether there were 1,862 potential pathogenic mutations located in 1,381 genes (Table S4), and 81 were overlapped with scoliosis related genes (Figure 1C). By comparing with randomized gene list, these potential pathogenic genes undergoing mutations were significantly enriched in scoliosis genes (*P* = 4.45 × 10^−07^). Since both of the parents had no spinal deformity, these potential pathogenic mutations may only increase the susceptibility of scoliosis without causing scoliosis directly.

**Table 1:** Different mutations in the MZ twin discordant for AIS.

**Type**	**Count**
Equal type (both mutation)	30,700
Different type 1 (both mutation)	32
Different type 2 (only mutation in Control)	0
Different type 3 (only mutation in Case)	1,830

### DNA Methylation Differences in the MZ Twins

**Figure 2:**
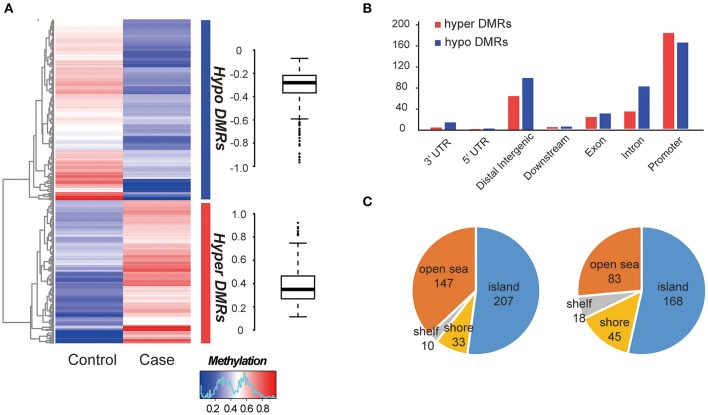
Comparison of DMRs between two samples. **(A)** Hierarchical clustering of DNA methylation profiles of AIS twins. The distribution of the methylation difference was shown in the box plot at the right side. **(B)** Characteristics of different functional element DNA methylation profiles of AIS twins. **(C)** CpG island distributions of hypomethylated DMRs (left) and hypermethylated DMRs (right).

The global methylation status between the MZ twins were very similar, only a small percentage of promoter existed difference (348/27967, 1.24%, Table S5). We compared whole-genome DNA methylation level between the MZ twins, and found that both the hyper- and hypo- DMRs were differently distributed (Figure 2A). Finally, we identified 313 hypermethylated DMRs and 397 hypomethylated DMRs (Table S5). The average hypermethylation difference was 38% and the average hypomethylation difference was 31% between the two samples. We found that more DMRs locate in the promoter regions (Figure 2B) and most of them located in CGI (CpG island) (Figure 2C). Significant methylation level difference between the twins may influence the function of DMR-related genes and contribute to the etiology of this disease. Interestingly, these DMRs overlapped with 25 allele-specific methylated regions, suggesting that they were related to abnormal genomic imprinting (Figure S3) (de Sa Machado Araujo et al., 2018).

**Figure 3:**
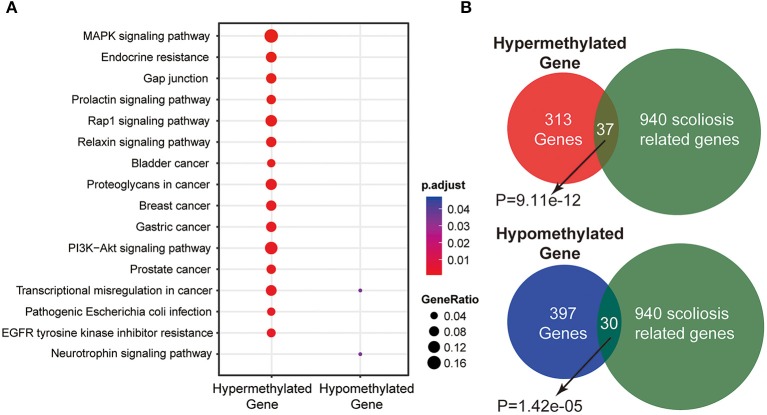
Annotations of DMR related genes between two samples. **(A)** Scatterplot of DMR related genes enrichment with KEGG pathway. **(B)** Enrichment of DMR related genes with scoliosis related genes.

We enriched the genes associated with these DMRs and then subjected these genes to KEGG (Kyoto Encyclopedia of Genes and Genomes) pathway analysis. It revealed that DMR associated genes were enriched in several pathways. Three pathways with the most significant association were the MAPK signaling pathway, PI3K-Akt signaling pathway and Rap1 signaling pathway (Figure 3A), indicating that these pathways could be associated with AIS.

### Enrichment of Scoliosis Related Genes in DMRs

To explore the relationship between these DMRs and scoliosis, we enriched the DMR related genes and found they were significantly overlapped with scoliosis related genes. In hypermethylated DMR associated genes group, 37 genes were overlapped with these scoliosis genes. In hypomethylated DMR associated genes group, 30 genes were overlapped with scoliosis related genes (Figure 3B). After comparing to randomized gene list, the DMR associated genes were significantly overlapped with scoliosis genes (*p* = 9.11 × 10^−12^ and 1.42 × 10^−05^, respectively), indicating that the methylation of scoliosis-related genes may be associated with the risk of AIS development.

### Validation of Candidate DMRs in the Replication Cohort

For further identification of specific pathogenic epigenetic variants, we selected 14 DMRs from the literature for validation based on the related gene's function in the replication cohort (Table 2). All of the 14 genes were related to scoliosis and manifested with high methylation difference in the MZ twins. We found the methylation level of *NDN*: TSS-DMR (chr15:23932133-23932304, hg19) was significantly differentiated in the replication cohort. The mean methylation level in the AIS group was significantly higher than the mean methylation level in the control group (3.78 × 10^−1^ and 3.64 × 10^−1^, *p* = 0.04). This DMR locates at the promoter region of gene *NDN*. The different methylation of this gene may influence gene expression and be associated with the phenotype of AIS.

**Table 2:** Selected epigenome DNAm difference region in the replicated cohort.

**Name of DMRs**	**Selected DMR position**	**Mean methylation level in AIS group**	**Mean methylation level in control group**	***P*-value**	***Z*-value**	**MethylDiff**
*CASK*:TSS-DMR	chrX:41783051-41783257	4.31 × 10^−1^	4.26 × 10^−1^	0.68	0.46	5.39 × 10^−3^
*EMD*:TSS-DMR	chrX:153606503-153607014	3.92 × 10^−1^	4.01 × 10^−1^	0.20	−0.84	−9.31 × 10^−3^
*ESR1*:TSS-DMR	chr6:152128858-152128958	2.98 × 10^−2^	3.14 × 10^−2^	0.74	0.64	−1.64 × 10^−3^
*FMR1*:TSS-DMR	chrX:146993416-146993736	4.28 × 10^−1^	4.17 × 10^−1^	0.83	0.95	1.06 × 10^−2^
*GDI1*:TSS-DMR	chrX:153665399-153665481	4.93 × 10^−1^	4.86 × 10^−1^	0.95	1.61	6.81 × 10^−3^
*GPC3*:TSS-DMR	chrX:133119090-133119453	4.01 × 10^−1^	3.98 × 10^−1^	0.43	−0.18	3.41 × 10^−3^
*KCNQ1OT1*:TSS-DMR	chr11:2720966-2721111	4.05 × 10^−1^	4.09 × 10^−1^	0.55	0.12	−4.21 × 10^−3^
*MAP2K2*:TSS-DMR	chr19:4123972-4124036	1.47 × 10^−2^	1.60 × 10^−2^	0.28	−0.59	−1.32 × 10^−3^
*NDN*:TSS-DMR	chr15:23932133-23932304	3.78 × 10^−1^	3.64 × 10^−1^	**0.04**	−1.75	1.45 × 10^−2^
*PAX1*:TSS-DMR	chr20:21687204-21687404	4.90 × 10^−2^	5.45 × 10^−2^	0.11	−1.24	−5.51 × 10^−3^
*PHF8*:TSS-DMR	chrX:54069169-54069423	4.85 × 10^−1^	4.67 × 10^−1^	0.54	0.09	1.77 × 10^−2^
*RPS6KA3*:TSS-DMR	chrX:20285105-20285182	3.17 × 10^−1^	3.23 × 10^−1^	0.33	−0.45	−6.05 × 10^−3^
*TUBB2B*:TSS-DMR	chr6:3227646-3227824	2.25 × 10^−2^	2.40 × 10^−2^	0.28	−0.59	−1.45 × 10^−3^
*TUBB3*:TSS-DMR	chr16:89989983-89990156	3.71 × 10^−2^	4.07 × 10^−2^	0.11	−1.20	−3.59 × 10^−3^

*The bold values mean statistically significant*.

## Discussion

This study represented the first combined analysis of whole exome and whole epigenome of MZ twins discordant for AIS. According to previous studies, genetic variations can predispose to AIS (Buchan et al., 2014a; Gao et al., 2017). Therefore, we first performed WES to detect genetic variations. We found three *de novo* SNVs, but the healthy twin sister also had these SNVs, indicating that pathogenesis of AIS may not be on account of these variants. Epigenetic variation is widely acknowledged to be involved in the pathogenesis of various diseases (Jin et al., 2014; Lyu et al., 2018). In this study, we compared whole-genome DNA methylation between the discordant twins using WGBS. Potential pathological DMRs were identified. The DMR related genes were enriched predominantly in the MAPK signaling pathway, and other 12 signaling pathways (i.e., PI3K-Akt and Rap1 signaling pathways etc.). The MAPK and PI3K-Akt signaling pathways have been reported to play an important role in osteoblast differentiation and skeletogenesis (Ge et al., 2007; Zhang et al., 2014; Iezaki et al., 2018). Previous studies also indicated that Rap1 signaling pathway is critical for myogenic differentiation and osteoclast function (Pizon et al., 1996; Zou et al., 2013). Abnormalities of these pathways could give rise to disorders manifested with scoliosis (Tiffin et al., 2013; Martinez-Lopez et al., 2017; Tsai et al., 2018). The previous study also showed that MAPK signaling pathway and PI3K-Akt signaling pathway were downregulated in bone marrow mesenchymal stem cells (BM-MSCs) of AIS, which may contribute to the AIS initiation and development (Zhuang et al., 2016). We hypothesized that hypermethylation of these DMR related genes could regulate MAPK, PI3K-Akt, and Rap1 signaling pathways, which may influence the initiation of AIS. This regulation mechanism may contribute to the discordance of AIS in this twin pair.

We also found the DMR associated genes were significantly overlapped with scoliosis related genes, which indicated that the methylation of scoliosis-related genes may play a pivotal role in the development of AIS. To explore specific epigenetic variants, we compared the specific methylation difference of several DMRs in the replication cohort. We found the *NDN*: TSS-DMR (chr15:23932133-23932304, hg19) was significantly differentiated in the replication cohort. The AIS group had higher methylation level of this DMR than the control group. This DMR locates in the promoter area of *NDN* and has 146 base pairs distance to TSS. *NDN* locates at 15q11.2 and is an intronless gene located in the Prader-Willi syndrome (PWS) deletion region. Previous studies hypothesized that lack of its coding protein Necdin during development may contribute to PWS (Jay et al., 1997; Miller et al., 2009). PWS is a rare disease associated with a variety of musculoskeletal abnormalities, about 43.4% of patients were afflicted with scoliosis (Odent et al., 2008). Besides, *NDN* is a well-known maternally imprinted gene and is expressed exclusively from the paternal allele, and its methylation are persistent markers of gene regulation (Lau et al., 2004). Previous studies had proved that imprinted DMRs (iDMRs) could be perturbed in kinds of diseases (de Sa Machado Araujo et al., 2018). In our study, we hypothesized that higher methylation could decrease the expression of *NDN*, which predisposed the patient to AIS.

There are several limitations in our study. First, since WES only covered most exons of the human reference genome, we did not assess the rest of the genome and other types of variants, such as karyotype or structural variants. Therefore, we cannot exclude the possibility that genome variants contribute to the etiology of this MZ twin pair discordant for AIS. Second, since all of the AIS patients were teenagers, the age of the control group is not matched with the case group in the replication cohort. The unmatched age may cause bias of the implications. Third, the sample size of the MZ twins and the replication cohort was relatively small. Larger samples are needed for the replication.

## Conclusion

In conclusion, we described the genome methylation difference in an AIS discordant MZ twin pair using WGBS and selected DMR methylation difference in a replication cohort. We found that the *NDN*: TSS-DMR had higher methylation level in AIS, which may elucidate the potential etiology of AIS.

## Data Availability Statement

The datasets generated for this study can be found in the http://discostudy.org/data/DISCO2019-1.html.

## Ethics Statement

The studies involving human participants were reviewed and approved by Ethical Review Board of Peking Union Medical College Hospital. Written informed consent to participate in this study was provided by the participants' legal guardian/next of kin.

## Author Contributions

GL, LW, and NW performed the research, analyzed, and interpreted the data. GL drafted the manuscript. XW, XY, ML, ZY, SL, YZu, YN, SZ, and YZh helped sample collection. JZ and JS performed phenotyping of patients. XW, ZY, and XY helped analysis and interpretation of NGS data. YN provided technique support. GQ, YW, ZW, JZ, and JS offered professional discussions and instructions. ZW and NW conceived and designed the study, revised the manuscript, and provided the final approval of the manuscript.

### Conflict of Interest

The authors declare that the research was conducted in the absence of any commercial or financial relationships that could be construed as a potential conflict of interest.
