# Supplementary material for: Whole-Genome Methylation Analysis of Phenotype Discordant Monozygotic Twins Reveals Novel Epigenetic Perturbation Contributing to the Pathogenesis of Adolescent Idiopathic Scoliosis
Source: Front Bioeng Biotechnol. 2019 Dec 10;7:364. doi: 10.3389/fbioe.2019.00364 (PMC6914696; doi:10.3389/fbioe.2019.00364)
Supplement: Supplementary file 6 [file Data_Sheet_1.docx]

# Supplementary Figures and Tables

## Supplementary Figures


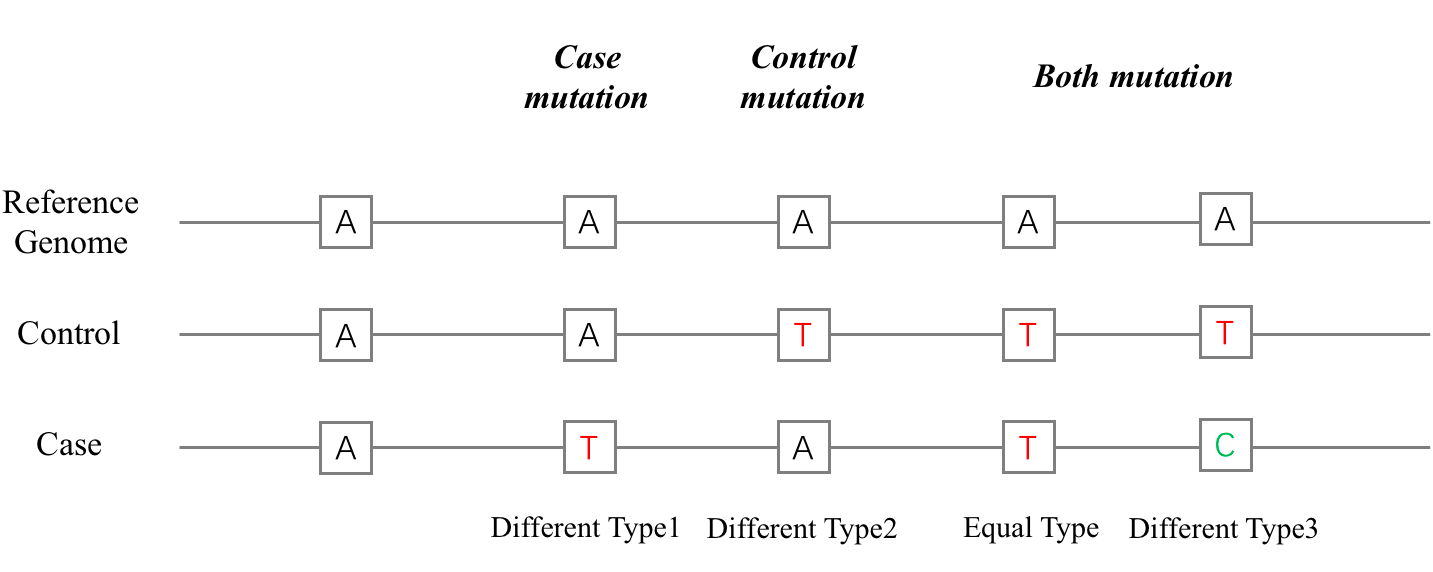


**Supplementary Figure 1.** Four types of mutations identified by Whole Exome Sequencing.


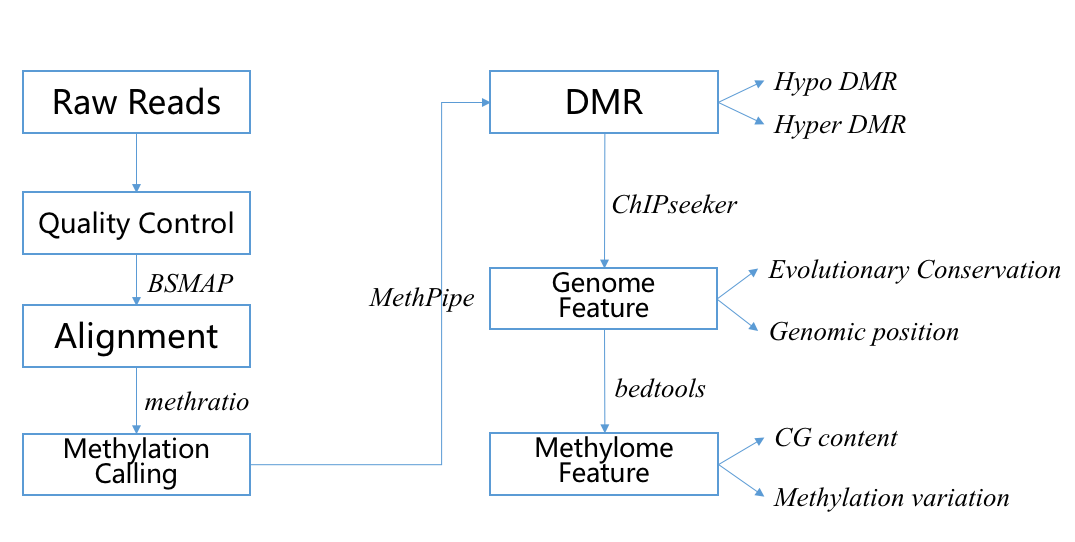


**Supplementary Figure 2.** Flowchart of Whole Genome Bisulfite Sequencing data analysis.


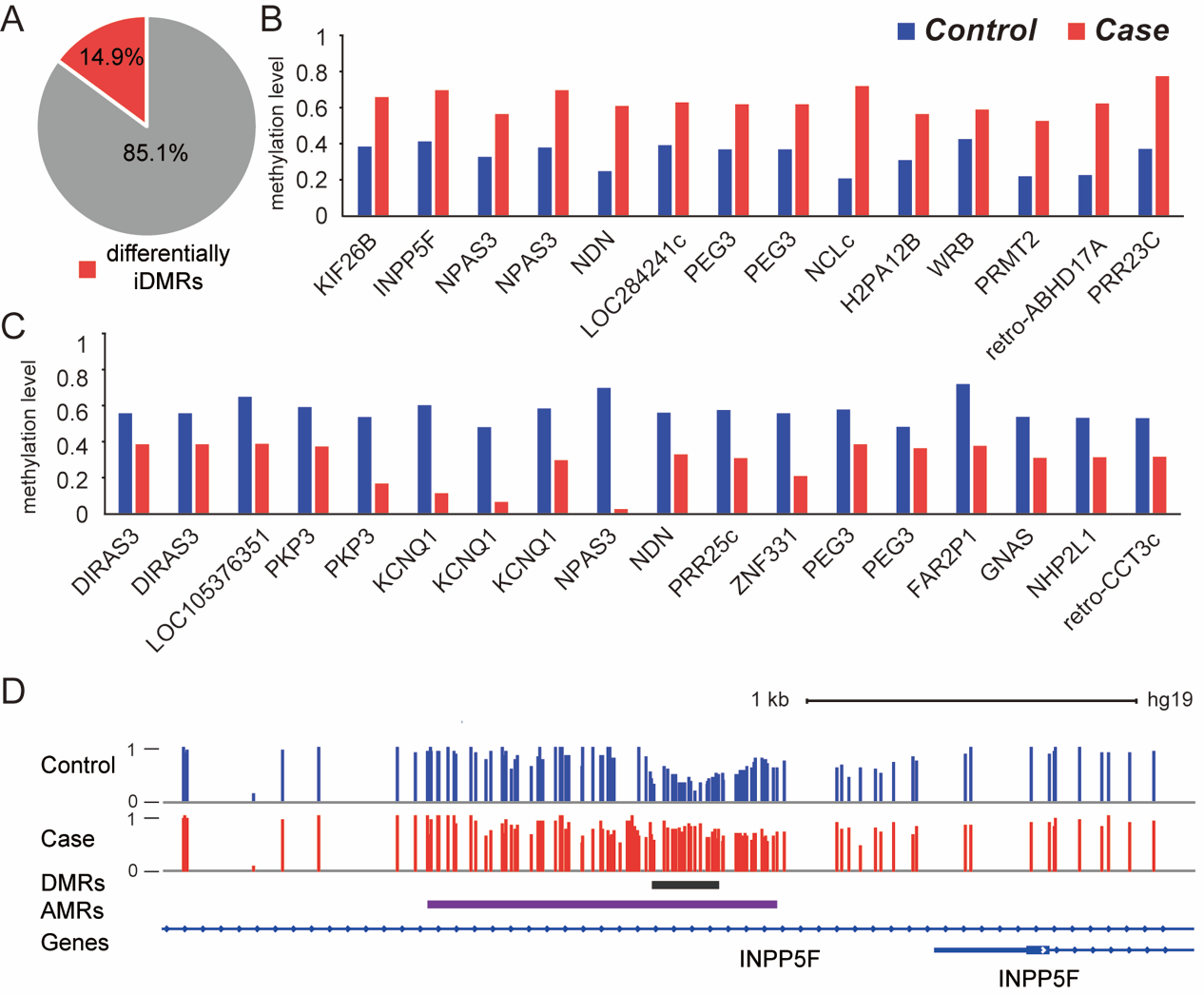


**Supplementary Figure 3.** Methylation status of iDMRs in the MZ twin samples.

## Supplementary Tables (see the attached excel profile)

**Table S1.** Whole Exome Sequencing quality control of the quad family.

**Table S2.** scoliosis related gene list.

**Table S3.** de novo mutations identified by Whole Exome Sequence in the MZ twins family.

**Table S4.** different mutations between the MZ twins.

**Table S5.** all Different Methylation Regions of Whole Genome Bisulfite Sequencing between the MZ twins.
